# Domestic work stress and self-rated psychological health among women: a cross-sectional study in Japan

**DOI:** 10.1186/s12199-019-0833-5

**Published:** 2019-12-17

**Authors:** Eri Maeda, Kyoko Nomura, Osamu Hiraike, Hiroki Sugimori, Asako Kinoshita, Yutaka Osuga

**Affiliations:** 1 Department of Environmental Health Science and Public Health, Akita University Graduate School of Medicine, Akita, Japan; 2 Department of Obstetrics and Gynecology, Graduate School of Medicine, The University of Tokyo, Tokyo, Japan; 3 Department of Preventive Medicine, Graduate School of Sports and Health Sciences, Daito Bunka University, Saitama, Japan

**Keywords:** Domestic work, Job strain, Demand-control-support model, Gender equity, WAFCS-J

## Abstract

**Background:**

Despite the huge burden of domestic work on women in Japan, its effects on their health have been poorly investigated. We aimed to assess the association between domestic work stress and self-rated psychological health among women.

**Methods:**

We conducted a cross-sectional survey using an online social research panel in February 2018. Participants were 2,000 women with paid work (the “workers” group) and 1,000 women without paid work (the “homemakers” group), aged between 25 and 59 years old and living with a partner. Self-rated psychological health (Mental Health and Vitality scales of the Japanese SF-36), occupational and domestic work stress (the Brief Job Stress Questionnaire), the 10-item Work–Family Conflict Scale, and sociodemographic factors were assessed.

**Results:**

The workers had lower domestic job control and higher support from a partner and their parents than the homemakers (*p* < 0.001), whereas domestic job demand and psychological health were similar between the groups. After adjustment for the covariates using multiple linear regression models, better psychological health was significantly associated with lower domestic job demand, higher domestic job control, and having a young child in both groups. In addition, work–family conflicts and occupational job stress among the workers and caregiving among the homemakers showed negative associations with psychological health.

**Conclusion:**

Self-rated psychological health in women was associated with domestic work stress regardless of employment status. To promote women’s health, we need to take into account the effects of domestic work, work–family conflicts, and social support from families, as well as occupational factors.

## Introduction

A growing number of women have joined the labor force in many countries over the past few decades. In Japan, the employment rate of women aged between 25 and 44 years has markedly increased from 57% in 1986 to 73% in 2016 [1]. In contrast, time spent in unpaid work for men has stayed extremely low (40.8 min per day) compared to that of the most advanced country with active participation of men in housework and childrearing (e.g., 171 min per day in Sweden) [2]. Even among married couples with small children, the average time spent for domestic work per day was only 83 min among husbands, which was much shorter than among wives, 454 min [1]. These results reflect the traditional gender roles of men being breadwinners and women being responsible for family that are entrenched in the Japanese mindset [3, 4]. In addition to the unequal domestic work division, Japanese women tend to complete domestic work within the family themselves. A recent governmental survey revealed that about 97% of women had never used professional homemaker service, not only because of the expense but also due to hesitation about letting other people enter their living space [5]. Also, less than half of women own dishwashers or drying machine [5]. Furthermore, the quality of domestic work is high; a Western newspaper has reported that typical Japanese dinner consists of multiple small dishes and that packed lunches that women prepare for children are “works of art.” [6] Thus, the quantity and quality of domestic work for Japanese women are likely tremendous.

In the face of a recent severe labor shortage, the government of Japan has started to make efforts to facilitate women’s active participation in the labor force as a major policy issue by encouraging men’s participation in domestic work and childrearing to enable women to join the workforce [7]. Women’s multiple roles as wives, mothers, and/or employees generally benefit their health by providing broad social networks, financial security, and self-esteem [8–10]. At the same time, liability related to domestic and occupational work has been conceptualized as a “double burden” [11, 12] that has significant associations with physical and mental disorders [13, 14]. A large body of literature identifies work–family conflicts [15] as a factor for adverse health outcomes among employed women [16], but few in-depth studies address the effects of domestic work itself on women’s health [17, 18]. Given the substantial amount of domestic work completed by women and differences in the nature of domestic work by culture or country [6, 19], we focused on domestic work stress among Japanese women, applying methods commonly used in occupational health evaluation, such as the job demand-control-support (DCS) model [20–23]. This approach may reveal the extent to which individual factors associated with domestic work affects self-perceived health.

Hence, our aim in this study was to investigate the relationship between stress associated with unpaid work at home and self-rated psychological health status of women living with a partner in Japan. Based on a previous study in Sweden [18], which first applied the demand-control model [24] to domestic work, we hypothesized that a domestic DCS model [21] would show the independent association of domestic work with psychological health even after adjusting for occupational factors, work–family conflicts, and sociodemographic factors in Japan. Specifically, we expected that a partner’s support would contribute to better psychological health by reducing the domestic workload for women.

## Methods

### Participants

**Fig. 1:**
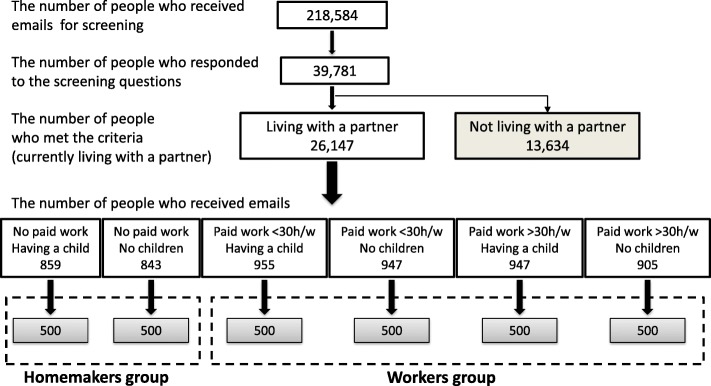
Flowchart of the procedure

Participants were recruited via an online social research panel (SRP). Inclusion criteria were being a woman aged between 25 and 59 years of age and living with a partner. We included two groups: women with paid work (the “workers” group, *N* = 2,000) and those without paid work (the “homemakers” group, *N* = 1,000). An online market research company (Macromill, Tokyo, Japan), which has a nationwide SRP of more than 1 million registrants, sent prescreening emails for the inclusion criteria to 218,584 people aged 25–59 years, who were randomly selected from its registrants (Fig. 1). Of the 26,147 eligible people, 5,456 people received recruitment emails and 3,000 people completed the survey (55% participation rate among eligible subjects). We performed quota sampling by age-group block (i.e., 25–29, 30–39, 40–49, and 50–59 age groups), paid work hours per week (i.e., no work, < 30 h, or ≥ 30 h), and whether a person had a child or not, according to the prescreening responses. Recruitment continued until the intended number of participants in each block had been recruited. Participants were provided a reward incentive consistent with the SRP procedure. All the procedures were completed from February 20 to 25, 2018.

### Measures

We developed a questionnaire to investigate the factors associated with women’s health in Japan. We conducted a pilot test of the survey with a small group of our female colleagues to ensure that the questionnaire was understandable.

### Self-rated psychological health

To measure psychological health of the participants, we used mental health and vitality scales of the Japanese SF-36, which were confirmed to be the best psychological health measures of the eight scales in the SF-36 in Japan [25]. The norm-based score (range 0–100) of each scale was calculated, standardizing scores to a normalized mean of 50 and standard deviation of 10, using population norms. Higher scores indicate better health-related quality of life.

### Occupational factors

We used the Brief Job Stress Questionnaire (BJSQ) [26] to evaluate job demand, job control, supervisor support, and coworker support for the workers group based on the DCS model [21]. The BJSQ has been widely used for occupational health and research in Japan [23, 27, 28]. Job demand consisted of three items: (i) you have to do an enormous amount of work; (ii) you cannot complete all your work in the allotted time; and (iii) you have to work very hard. Job control included three items: (i) you can work at your own pace; (ii) you can decide the order in which you do your work and the way you do it; and (iii) you can provide your opinions on the work strategy of your workplace. Supervisor support and coworker support were evaluated with the following items: (i) you can often communicate with supervisors/coworkers; (ii) you can strongly rely on supervisors/coworkers when you have problems; and (iii) your supervisors/coworkers are prepared to spend their time on your personal problems. Participants rated their level of agreement for each item on a 4-point scale from 1 (strongly disagree) to 4 (strongly agree). A higher summed score for each dimension (range 3–12) indicates higher job demand, more job control, higher supervisors’ support, or higher coworkers’ support. In addition, we included paid work hours per week in occupational factors.

### Domestic work factors

We applied the DCS model [21] to the measurement of domestic work stress. All the participants scored domestic work demand (three items with corresponding wordings to job demand measured with the BJSQ) and domestic work control (three items with corresponding wordings to job control measured with the BJSQ) on a 4-point scale from 1 (strongly disagree) to 4 (strongly agree). With regard to domestic work support, participants rated the level of support from a partner and parents or parents-in-law with a 4-point scale from 1 (poor support) to 4 (excellent support), instead of direct application of the job support items on the BJSQ. We dichotomized the level of supports: low (1 or 2) or high (3 or 4). In addition, we included as domestic work factors whether they provided care for any of their family members (i.e., unpaid caregiving, yes/no) and whether they had a young child (≤ 12 years old, yes/no).

### Work–family conflict

The Work–Family Conflict Scale (WAFCS) is a brief 10-item scale of work–family conflict comprising two subscales: work-to-family conflict (WFC, five items) and family-to-work conflict (FWC, five items) [29]. Participants in the workers group were asked to rate their level of agreement for each item with a 7-point scale from 1 (very strongly disagree) to 7 (very strongly agree). Scores are reported as sums of the points, providing the total WFC score (range 7–35) and FWC score (range 7–35). Higher scores indicate higher levels of conflict. Both subscales had good internal consistency (> 0.90) and construct validity, as well as concurrent and predictive validity [29]. The WAFCS, originally developed to examine work–family conflict in parents of young children, has been applied to a variety of participants by defining “family” from the participant’s perspective [30].

We developed the Japanese version of the WAFCS (WAFCS-J) using forward translation and back translation of the original WAFCS. Two professional translators conducted forward translations separately, and two Japanese public health researchers (EM and HS) merged two translations into one. A bilingual Japanese–English individual translated the resulting questionnaire back into English. We confirmed the concordance between the back-translated items and the originals after minor changes were made, in line with discussion between the Japanese researcher (EM) and the developer of the original WAFCS [29]. To evaluate reliability and validity of the WAFCS-J, we calculated internal consistency coefficient alpha (coefficient alpha = 0.85 for WFC and 0.89 for FWC) and conducted a factor analysis with promax rotation (Table 4 in Appendix). The eigenvalues of the factors were 4.95 and 1.01 (> 1.0, the Kaiser’s criteria of eigenvalues) [31], which accounted for 86% and 18% of the variance, respectively. All the items had high factor loadings after promax rotation (≥ 0.40) [32] on the designated factors. Thus, the WAFCS-J appeared to have a two-factor structure and good internal consistency, as did the original WAFCS.

### Sociodemographic factors

Age in years and categorized annual household income of the participants were provided by the online market research company. Annual household income was categorized into four groups: low, < 4 million Japanese Yen (JPY); moderate, 4–7 million JPY; high, ≥ 8 million JPY; and “unknown.” Participants reported their academic background (university education, yes/no).

### Statistical analyses

We compared the characteristics between the workers group and the homemakers group, using Student *t* tests, chi-square tests, and Fisher exact tests, depending on the type and distribution of the variables.

We performed the following analyses separately by groups. We compared self-rated psychological health scores between categories, using Student *t* tests or one-way analysis of variance as appropriate. We used Pearson correlation analyses to examine the univariate association between self-rated psychological health scores and the level of job demand and control (domestic and occupational) and occupational support from supervisor and coworkers. To assess the association between self-rated psychological health and domestic work stress, we conducted multiple linear regression analyses controlling for other covariates based on previous studies [13, 14, 17, 23, 33]. We calculated the robust estimator of variance (White-corrected standard errors) [34], considering the heteroscedasticity of the variables. Additionally, we conducted subgroup analysis in the workers group by working hours per week (i.e., < 30 h or ≥ 30 h).

A two-sided *p* value of < 0.05 was used to define statistical significance. All analyses were performed using Stata14-MP (StataCorp LP, College Station, TX, USA).

## Results

Table 1 shows the characteristics of the two groups. Participants were about 40 years of age and of low or middle income, with about one third having a university education. In the workers group, mean working time was 30.7 h per week; 40 women (2.0%) in this group reported 60 h or more per week. Those in the workers group were more likely to be a smoker or a habitual drinker and to have university education, higher household income, lower domestic job control, and higher support from a partner and their parents. Domestic job demand was similar between the groups.

**Table 1:** Characteristics and self-rated psychological health of the study participants

	Workers (*N* = 2,000)	Homemakers (*N* = 1,000)	*p* value
Demographics			
Age in years, mean ± SD	41.8 ± 9.6	41.9 ± 9.9	0.83
University education, *N* (%)	698 (34.9)	304 (30.4)	0.01
Annual household income, *N* (%)			< 0.001
Low: < 4 million JPY	360 (18.0)	214 (21.4)	
Middle: 4–7 million JPY	793 (39.6)	369 (36.9)	
High: ≥ 8 million JPY	379 (19.0)	134 (13.4)	
Unknown	468 (23.4)	283 (28.3)	
Lifestyles			
Current smoker, *N* (%)	329 (16.4)	105 (10.5)	< 0.001
Habitual drinker (once a week or more), *N* (%)	605 (30.2)	223 (22.3)	< 0.001
Domestic work factors			
Job demand, mean ± SD [range: 3–12]	7.5 ± 1.9	7.5 ± 1.9	0.95
Job control, mean ± SD [range: 3–12]	9.5 ± 1.8	9.9 ± 1.7	< 0.001
High support from a partner, *N* (%)^a^	765 (38.2)	295 (29.5)	< 0.001
High support from parents or parents-in-law, *N* (%)^a^	402 (20.1)	141 (14.1)	< 0.001
Having a young child, *N* (%)	564 (28.2)	308 (30.8)	0.14
Giving care to a family member, *N* (%)	96 (4.8)	58 (5.8)	0.24
Occupational factors			
Job demand, mean ± SD [range: 3–12]	7.5 ± 2.1	–	
Job control, mean ± SD [range: 3–12]	8.1 ± 2.2	–	
Support from supervisors, mean ± SD [range: 3–12]	7.1 ± 2.4	–	
Support from co-workers, mean ± SD [range: 3–12]	7.8 ± 2.4	–	
Working hours per week, mean ± SD	30.7 ± 14.1	–	
Work–family conflicts			
Work-to-family conflicts, median (IQR) [range: 5–35]	16 (10–20)	–	
Family-to-work conflict, median (IQR) [range: 5–35]	10 (5–16)	–	
Self-rated psychological health on the SF-36			
Mental Health score	46.3 ± 10.1	46.6 ± 10.7	0.45
Vitality score	44.8 ± 10.2	45.4 ± 10.5	0.10

*p* values were calculated by Student *t* test for continuous variables and chi-square tests for categorical variables

IQR, interquartile range

^a^Those who received excellent or good support

The means ± SD of self-rated psychological health scores of Japanese *SF-36* were similar between the groups (Table 1). Univariate associations between self-rated psychological health scores and associated factors are shown in Table 2. In the workers group, the Mental Health score showed negative correlations with domestic and occupational job demand and work–family conflicts, and positive correlations with domestic and occupational job control and occupational support from supervisors and co-workers. The Mental Health score was significantly higher among participants in their 50s than among those in their 20s and was higher among participants with middle/high/unknown household income than among those with low household income. Multiple linear regression analyses showed that a higher Mental Health score was associated with lower domestic job demand, higher domestic job control, lower occupational job demand, higher occupational job control, higher occupational support from supervisors and co-workers, lower work–family conflicts, being in their 50s, having a young child, and having higher household income. Similar univariate and multivariate associations between the Vitality score and the study variables were observed. Additionally, having high support from parents or parents-in-law was positively associated with the Vitality score.

**Table 2:** Univariate analyses and multiple linear regression models for factors related to the SF-36 health scores by group

	Workers group (*N* = 2,000)								Homemakers group (*N* = 1,000)							
	Mental Health score				Vitality score				Mental Health score				Vitality score			
	Univariate		Multivariate *R*^*2*^ = 0.24		Univariate		Multivariate *R*^*2*^ = 0.20		Univariate		Multivariate *R*^*2*^ = 0.18		Univariate		Multivariate *R*^*2*^ = 0.17	
	Mean ± SD or *r*	*P*	*B*	*P*	Mean ± SD or *r*	*P*	*B*	*P*	Mean ± SD or *r*	*P*	*B*	*P*	Mean ± SD or *r*	*P*	*B*	*P*
Domestic work factors																
Job demand	− 0.21 ^a^	<0.01	-0.37	<0.01	− 0.19 ^a^	<0.01	− 0.31	0.02	− 0.29 ^a^	< 0.01	-1.33	<0.01	-0.26 ^a^	<0.01	− 1.05	<0.01
Job control	0.22 ^a^	<0.01	0.34	<0.01	0.18 ^a^	<0.01	0.29	0.03	0.29 ^a^	< 0.01	1.49	<0.01	0.29 ^a^	<0.01	1.48	<0.01
Support from a partner		0.09 ^b^				<0.01 ^b^				0.04 ^b^				0.02 ^b^		
No	46.0 ± 10.2		Ref		44.3 ± 10.2 ^b^		Ref		46.2 ± 10.5		Ref		44.9 ± 10.3		Ref	
Yes	46.8 ± 10.0 ^b^		-0.16	0.71	45.6 ± 10.2		0.31	0.49	47.7 ± 11.2		0.50	0.50	46.6 ± 10.9		0.45	0.53
Support from parents or parents-in-law		0.07 ^b^				<0.01^b^				0.20 ^b^				0.02 ^b^		
No	46.1 ± 10.0		Ref		44.3 ± 10.1		Ref		46.4 ± 10.7		Ref		45.1 ± 10.5		Ref	
Yes	47.1 ± 10.5 ^b^		0.71	0.19	46.7 ± 10.4 ^b^		1.87	<0.01	47.7 ± 11.1		1.12	0.25	47.4 ± 10.4		2.23	0.02
Having a young child		0.74 ^b^				0.69 ^b^				0.21 ^b^				0.76 ^b^		
No	46.3 ± 10.2		Ref		44.8 ± 10.4		Ref		46.3 ± 11.0		Ref		45.4 ± 10.8		Ref	
Yes	46.2 ± 10.0 ^b^		1.81	<0.01	44.6 ± 9.7 ^b^		1.52	<0.01	47.2 ± 10.1		2.68	<0.01	45.6 ± 9.8		1.62	0.02
Providing care for a family member		0.45 ^b^				0.50 ^b^				< 0.01 ^b^				< 0.01^b^		
No	46.3 ± 10.1		Ref		44.8 ± 10.2		Ref		46.9 ± 10.6		Ref		45.8 ± 10.3		Ref	
Yes	45.5 ± 9.4 ^b^		-0.24	0.81	44.1 ± 10.0 ^b^		− 0.63	0.54	41.3 ± 11.8		-4.96	<0.01	39.8 ± 12.1		-5.64	<0.01
Demographic factors																
Age group										0.48 ^c^				0.06 ^c^		
25–29	45.3 ± 10.3	Ref	Ref		44.2 ± 10.4	Ref	Ref		47.2 ± 11.0		Ref		47.0 ± 10.4		Ref	
30–39	46.3 ± 10.0 ^c^	0.92 ^c^	0.97	0.14	44.9 ± 9.7 ^c^	1.00 ^c^	0.81	0.23	46.0 ± 11.1		-0.70	0.52	44.4 ± 10.0		-2.06	0.04
40–49	45.6 ± 10.5 ^c^	1.00 ^c^	0.66	0.34	44.0 ± 10.5 ^c^	1.00 ^c^	0.34	0.63	46.3 ± 10.4		-0.66	0.54	45.0 ± 11.3		-1.72	0.10
50–59	47.4 ± 9.6 ^c^	0.02 ^c^	1.70	0.02	45.6 ± 10.2 ^c^	0.35 ^c^	1.25	0.11	47.2 ± 10.6		0.87	0.46	46.1 ± 10.2		-0.11	0.92
University education		0.28 ^b^				0.34 ^b^				0.10 ^b^				0.49 ^b^		
No	46.1 ± 10.2		Ref		44.6 ± 10.3		Ref		46.2 ± 10.8		Ref		45.3 ± 10.8		Ref	
Yes	46.6 ± 10.0 ^b^		0.35	0.42	45.1 ± 10.0 ^b^		0.40	0.38	47.5 ± 10.5		0.75	0.27	45.8 ± 9.9		0.02	0.97
Household income																
Low	43.7 ± 11.1	Ref	Ref		43.6 ± 11.1	Ref	Ref		44.8 ± 11.8	Ref	Ref		43.2 ± 11.5	Ref	Ref	
Middle	46.4 ± 9.8 ^c^	<0.01	2.10	<0.01	44.3 ± 10.1 ^c^	1.00 ^c^	0.25	0.70	46.2 ± 10.1	0.77^c^	1.45	0.11	44.9 ± 10.0	0.37 ^c^	1.85	0.04
High	48.0 ± 9.5 ^c^	<0.01	3.20	<0.01	46.0 ± 9.7 ^c^	<0.01 ^c^	1.62	0.03	49.5 ± 9.9	< 0.01^c^	4.03	<0.01	47.9 ± 10.2	<0.01	4.35	<0.01
Unknown	46.8 ± 9.9 ^c^	<0.01	2.19	<0.01	45.4 ± 9.9 ^c^	0.07 ^c^	1.09	0.11	47.2 ± 10.8	0.07 ^c^	2.01	0.04	46.7 ± 10.2	<0.01	3.15	<0.01
Occupational factors																
Job demand	− 0.22 ^a^	<0.01	-0.33	<0.01	− 0.21 ^a^	<0.01	− 0.37	<0.01								
Job control	0.19 ^a^	<0.01	0.26	0.02	0.18 ^a^	<0.01	0.28	<0.01								
Support from supervisors	0.25 ^a^	<0.01	0.40	<0.01	0.22 ^a^	<0.01	0.33	<0.01								
Support from co-workers	0.26 ^a^	<0.01	0.45	<0.01	0.22 ^a^	<0.01	0.39	<0.01								
Working hours per week		0.94 ^b^				0.05 ^b^										
< 30 h	46.3 ± 10.1		Ref		45.2 ± 9.9		Ref									
≥ 30 h	46.3 ± 10.1 ^b^		0.78	0.06	44.3 ± 10.4 ^b^		0.15	0.73								
Work–family conflicts																
Work-to-family conflicts	− 0.36 ^a^	<0.01	-0.28	<0.01	− 0.36 ^a^	<0.01	− 0.39	<0.01								
Family-to-work conflicts	− 0.34 ^a^	<0.01	-0.24	<0.01	− 0.25 ^a^	<0.01	− 0.02	0.62								

^a^*r,* Pearson correlation coefficient. ^b^Student *t* tests. ^c^One-way analysis of variance and Bonferroni multiple-comparison tests

Ref, reference; *R*^2^, coefficients for determination; *CI,* confidence interval; *B,* regression coefficient

In the homemakers group (Table 2), the Mental Health score was negatively correlated with domestic job demand and positively correlated with domestic job control. The Mental Health score was significantly higher among individuals who received high support from a partner and among those who had high household income, and lower among those who provided care for a family member. Multiple linear regression analyses showed that a higher Mental Health score was associated with lower domestic job demand, higher domestic job control, having a young child, having high household income, and not providing care for a family member. Similar univariate and multivariate associations between the Vitality score and the study variables were observed. Additionally, having high support from parents or parents-in-law was positively associated with the Vitality score.

Subgroup analyses by working hours revealed that lower domestic job demand and having a young child were positively associated with psychological health, irrespective of working hours (Table 3). Higher domestic job control among short-time workers (< 30 h per week) and high support from parents or parents-in-law among long-time workers (≥ 30 h per week) showed significant associations with psychological health scores, but there were no significant interactions.

**Table 3:** Subgroup analyses of regression coefficient *B* ^a^ for domestic work factors to self-rated mental health by working hours

	< 30 h per week (*N* = 1,000)				≥ 30 h per week (*N* = 1,000)			
	Mental Health score		Vitality score		Mental Health score		Vitality score	
	*B*	*P*	*B*	*P*	*B*	*P*	*B*	*P*
Domestic job demand (each additional point higher)	− 0.42	0.03	− 0.31	0.10	− 0.35	0.04	− 0.32	0.09
Domestic job control (each additional point higher)	0.41	0.03	0.38	0.04	0.24	0.16	0.18	0.35
High support from a partner	0.001	0.99	0.54	0.41	− 0.24	0.67	0.16	0.80
High support from parents or parents-in-law	0.06	0.94	0.97	0.22	1.23	0.09	2.64	< 0.01
Having a young child	2.22	< 0.01	1.35	0.06	1.50	0.04	1.77	0.02
Giving care to a family member	0.31	0.84	− 0.82	0.57	− 0.92	0.48	− 0.47	0.75

^a^Adjusted for demographic factors, occupational factors, and work–family conflicts using multiple linear regression models used in Table 2 (excluding working hours)

## Discussion

This quantitative study conducted in Japan is the first to demonstrate the independent relationship between domestic work stress and self-rated psychological health in Japan. We confirmed that higher domestic job demand and lower domestic job control are significantly associated with poorer self-rated psychological health both in the workers group and in the homemakers group. Similar regression coefficients of domestic and occupational job stress in the workers group suggest the importance of accounting for domestic factors when encouraging women to work in society. Based on the fact that the proportion of households with full-time homemakers is declining but still 38% in Japan [1], determining the domestic work stress for homemakers is important, particularly since their health has not received much attention in the past. In addition, we found no significant association between partners’ support and psychological health even after adjusting for covariates, whereas having a child or support from parents/parents-in-law showed positive associations with self-rated psychological health.

Negative associations between domestic workload and self-rated psychological health are consistent with previous studies. A higher domestic workload was reported to be associated with increased psychological distress [14, 35, 36], suboptimal self-rated health [17], and cardiovascular health risks [13, 37]. However, previous studies assessed domestic workload using a variety of indicators, such as domestic working hours [11, 12, 17, 36, 37], the unequal division of domestic work [14, 33, 35], subjective burden [17], and the factors constituting domestic work (e.g., childcare, living with the elderly) [13, 38]. Because each individual would define their own domestic work differently in terms of quality and quantity [19], we applied the DCS model [21] as a means to determine subjective domestic workload, and we confirmed the findings from the previous Swedish study [18]. Given these results and the lack of validated scales for domestic workload, applying the DCS model to domestic work is a promising method to quantify the psychological stress associated with domestic work among women. However, significant associations between domestic job control and psychological health were not found among women working for 30 h or more (Table 3), whereas domestic job demand consistently showed significantly negative associations with self-rated psychological health. We speculate that full-time workers might have a different view of domestic work, such as not giving importance to feeling in control of domestic work. Identifying factors and items contributing to high domestic job demand and low domestic job control would be an area for future research.

We did not detect significant associations between support from a partner and self-rated psychological health when we adjusted for covariates. Our analytical models confirmed that no collinearity existed between support from a partner/parents and having a child. Previously, marriage or support from a partner was shown to be a protective factor for overall subjective health of women [39]. Alternatively, the literature suggests that unequal division of domestic work and family responsibility is associated with less life satisfaction, poor self-rated health [40], and perceived physical/psychosomatic symptoms [14]. In this regard, the insignificant effect of support from a partner in the present study could be due to the study design; we excluded potential participants who had been divorced or were living separately, which might have decreased the statistical power to detect significant differences.

On the other hand, the insignificant results regarding support from a partner could illustrate the characteristics of married couples in Japan. First, the present participants might not have expected their partner to do much domestic work. Previous studies showed that women in societies in which traditional gender roles are followed did not recognize the unequal division of domestic work as an unfair situation [41]. Indeed, a fairly high percentage of the participants rated the level of support from a partner as “good” or “excellent” (35%, Table 1), in comparison with the national surveys indicating that men have extremely little unpaid work time [1]. Second, some support provided by a partner might not be sufficient to increase the level of satisfaction (i.e., psychological health) compared to the amount of domestic work routinely provided by women. Based on previous works showing higher mortality rates and risk-promoting dietary habits among unmarried men [42] and men living apart from their wives [43], men would directly benefit from their wives through the receipt of domestic work as well as social support. In contrast, mismatches between women’s expectation and domestic work actually completed by men have been pointed out [44]. To decrease domestic job stress, both wives and husbands are encouraged to remove traditional gender roles at home; in addition, discussing the social norms for domestic work and redefining them are necessary steps because the quantity and quality of the work have been determined by social, cultural, emotional, and environmental contexts [19]. It should be acknowledged that the idea of domestic work done to perfection held in the minds of Japanese women [6, 44] might have hampered the domestic participation of men and the equal division.

Having young children consistently showed positive associations with self-rated psychological health in all subgroups. Childrearing generally increases domestic workload and is known to increase the risk of poor self-rated health and fatigue among employed women, especially for those working long hours [45]. However, recent studies have shown that the presence of children, serving as a social support, had a mitigating effect on psychological burnout among academic professionals [46]. In this study, self-rated psychological health of the 628 women whose youngest child was older than 12 did not differ from that of women having no children (data not shown). This outcome could be explained by all the participants at least living with their partner and receiving some social support. Along with the positive associations between high support from parents/parents-in-law and Vitality scores, social support from families likely improves the psychological health of women.

Unpaid caregivers in the homemaker group showed significantly lower psychological health scores (Table 2). Substantial literature shows that caregivers generally report higher level of distress and depressive symptoms than non-caregiving peers [47]. At the same time, the extent of the psychological impact is affected by many factors, such as the type of disability of the recipient, the caregiver’s financial situation, the quality and quantity of social support, and whether or not the caregiver lives with the recipient [47]. Although health status or disability of the recipient could have been more serious in the homemakers group than in the workers group, social support at work places, income obtained from occupational work, or the use of professional care services might have alleviated the burden of caregiving.

We confirmed negative associations between occupational job stress and psychological health (Table 2) in line with previous studies using the DCS models [23, 27]. However, no significant relationships were found between working hours and self-rated psychological health, possibly because only 2% of the present workers worked for 60 h or more per week. Earlier studies showed significant associations between working hours and depressive symptoms or burnout [23, 48] only when the working hours exceeded 60 h, which would be consistent with our finding of insignificant results. Multiple roles of women generally restrict their working hours as employees. According to government statistics, the proportions of Japanese men and women who work for 60 h or more are 11% and 2.8%, respectively [49]. Occupational job demand measuring the subjective amount of work in a set period of time would be appropriate for measuring the workload of women or others who work in diverse ways [50].

Another contribution of the present study is the development of the WAFCS-J. A number of Work–Family Conflict Scales that vary in construction (e.g., from one to six dimensions) and in length (e.g., from 2 to 22 items) have been developed [51, 52], but only a few validated scales that are brief and sensitive exist. The WAFCS, which consists of 10 items, can easily be used as a secondary indicator in surveys [29]. A strength of the WAFCS is that it could be applicable not only for parents of small children but also for a wide range of people [30] in diverse family structures and lifestyles. Along with previous reports on the adverse health outcomes of work–family conflicts [16, 53], the present findings (Table 2) add evidence for the need to consider work–family conflicts during routine psychological health evaluations of employees.

This study has some limitations. First, it is a cross-sectional study, thus we cannot infer causality. Second, the use of an SRP could have caused selection bias. In particular, the percentage of the participants who had a university education was twice as high as that found in the 2010 Population Census (15% in women aged between 25 and 59 years old). Therefore, generalizing our results to the national population might cause self-rated psychological health to be overestimated. However, the association between domestic workload and self-rated psychological health shown in the multivariate linear regression models would not be heavily biased.

## Conclusion

Higher domestic job demand and lower domestic job control were associated with poorer self-rated psychological health among women with and without paid work in Japan. Health promotion of female workers requires consideration of the effects of domestic work, work–family conflicts, and the presence of social support from families. Future study is warranted for developing strategies to reduce domestic work stress, not only by removing the traditional gender roles at home but also by changing social norms related to domestic work.

## Data Availability

The datasets generated and/or analyzed during the current study are not publicly available due to including the privacy of the participants but are available from the corresponding author on reasonable request.

## Appendix

**Table 4:** Results of the exploratory factor analysis for the Japanese version of the Work–Family Conflict Scale with promax rotation

		Factor loadings		Uniqueness
		Factor 1 FWC *α* = 0.89	Factor 2 WFC *α* = 0.85	
1	My work prevents me spending sufficient quality time with my family 仕事のせいで、家族との充実した時間を十分にとれない。	− 0.04	**0.84**	0.33
2	There is no time left at the end of the day to do the things I’d like at home (e.g., chores and leisure activities) 一日の終わりに、家でやりたいこと(いろいろな用事や余暇活動など)をする時間が残っていない。	− 0.09	**0.70**	0.58
3	My family misses out because of my work commitments 私が仕事に専念するので、家族がさびしがる。	0.17	**0.55**	0.55
4	My work has a negative impact on my family life 仕事が家庭生活に悪影響を及ぼしている。	0.09	**0.77**	0.30
5	Working often makes me irritable or short tempered at home 仕事のせいで、家でイライラしたり、かっとなったりすることが多い。	0.11	**0.65**	0.50
6	My work performance suffers because of my personal and family commitments 私用や家庭の用事のために、仕事の成果が上がらない。	**0.66**	0.22	0.34
7	Family related concerns or responsibilities often distract me at work 家庭の心配事や用事のために、仕事に集中できないことが多い。	**0.64**	0.22	0.37
8	If I did not have a family I’d be a better employee 家族がいなければもっと優秀な社員になれると思う。	**0.76**	− 0.04	0.46
9	My family has a negative impact on my day to day work duties 家族は日々の仕事に悪影響を与えている。	**0.89**	− 0.12	0.31
10	It is difficult to concentrate at work because I am so exhausted by family responsibilities 家のことで疲れ果てて仕事に集中するのが難しい。	**0.79**	0.07	0.30

Factor loadings ≥ 0.40 are in bold

*FWC* family-to-work conflict; *WFC* work-to-family conflict; *α* Cronbach coefficient alpha
